# Anorectal malformation with long perineal fistula: one of a special type

**DOI:** 10.1038/s41598-021-81056-3

**Published:** 2021-01-18

**Authors:** Sen Li, Jun Wang

**Affiliations:** 1 Department of Pediatric Surgery, Xinhua hospital affiliated to Shanghai Jiao Tong University School of Medicine, No. 1665, Kongjiang Road, 200092 Shanghai, China; 2 Department of Pediatric Surgery, Shanghai Children′s Hospital, Shanghai Jiao Tong University, No. 355, Luding Road, 200000 Shanghai, China

**Keywords:** Paediatric research, Digestive signs and symptoms

## Abstract

The anorectal malformation with long perineal fistula is a rare anomaly in the spectrum of anorectal malformations. Aim of the study is to describe the series of patients with anorectal malformation with long perineal fistula and compare the outcome with patient with standard perineal fistula. From March 2012 to January 2019, 7 patients who suffered from anorectal malformation with long perineal fistula were retrospectively reviewed. Three were operated on primarily by our department, and 4 cases were re-operated after a perineal anoplasty repair performed elsewhere. Four were operated by laparoscopy assisted anorectoplasty, and 3 cases were repaired by posterior sagittal anorectoplasty. The follow-up outcomes were compared with 71 cases of normal perineal fistula (NPF) in the same period. 7 cases have been followed up for 0.5–4 years (M = 2.57 ± 1.26) after definitive surgery. Their bowel function score was lower than normal perineal fistula (SPF = 12, range: 5–18; NPF = 18.5, range: 18–20). Four cases underwent anorectomanometry. The incidence of rectoanal inhibitory reflex was lower in the special type group. (*p* = 0.14). Three cases of contrast enema using barium: 2 cases of colorectal dilatation and thickening changes, 1 case showed no obvious abnormalities. Anorectal perineal fistula should be examined by distal colostogram at preoperation. This should be altered in: When suspecting a case of anorectal malformation type long perineal fistula a preoperative contrast enema could give insight of the anatomy befor performing a anoplasty.

## Background

Perineal fistula is one of the most common types of anorectal malformation in pediatric surgery. Most pediatric surgeons agree that this well recognized malformation can be repaired by perineal anoplasty^1–3^.

However, we experienced a rare case of a perineal fistula associated with dilatation of the bowel found at a high position. This special type of malformation (SPF) is characterized by a perineal fistula as the principal clinical manifestation but the fistula tube is slender and the proximal bowel is found at a high position. Therefore, if a full preoperative assessment is not performed, the diagnosis may be delayed leading to abnormal bowel function.

As Peña’s paper in 2017 emphasized, a distal colostogram is the single most important diagnostic study indicated in children with an imperforate anus prior to definitive repair^4^. The aim of this article is to explore the diagnosis and treatment of this special type of perineal fistula, and to improve its treatment outcomes.

## Methods

From March 2012 to January 2019, seven patients who had a anorectal malformation with a special type of perineal fistula were either admitted or transferred to Department of Pediatric Surgery, Xinhua Hospital Affiliated to Shanghai Jiao Tong University School of Medicine. There are six boys and one girl.

**Figure 1:**
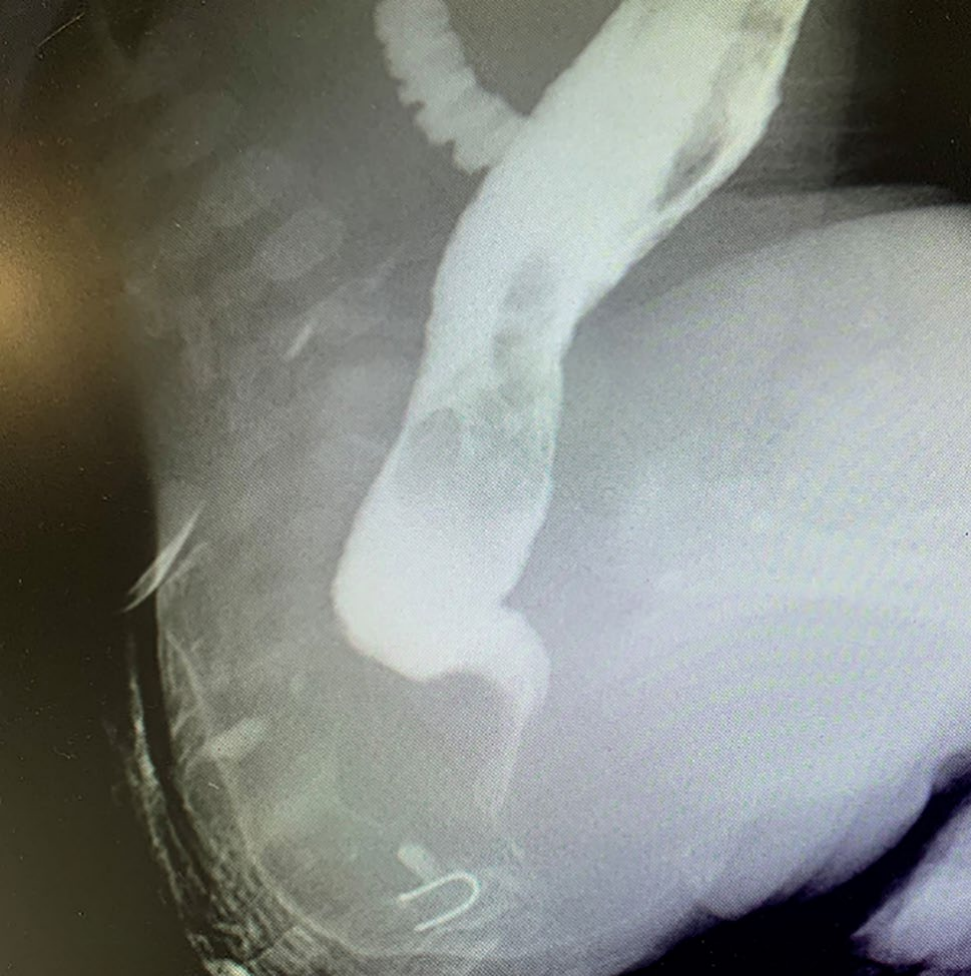
Colostogram showed the high position.

Among these patients, four were diagnosed with low rectal perineal fistula and had perineal anoplasty without undergoing a distal colostogram at a local hospital after birth. However, during the surgery, it was difficult to find the high positioned rectum, and therefore, the operation was terminated. After the perineum was sutured, a colostomy was performed. Thus, these patients only underwent colostomy before they were transferred to our institute after a period of three months. We performed a preoperative distal colostogram, which revealed that the fistula tube was slender, and the dilated proximal colon was located in front of the iliac crest (Fig. 1).

One patient was diagnosed with a rectal perineal fistula at the local hospital, and was treated with daily calibration. When she was 10 months old, she developed anal bleeding after anal sphincter treatment, and was subsequently transferred to our hospital. On the basis of a preoperative distal colostogram and magnetic resonance imaging (MRI), the patient was diagnosed with SPF, and initially underwent transverse colostomy.

The two other patients who came to our hospital after birth were diagnosed with SPF by a preoperative distal colostogram similar to the above case. Subsequently, colostomy was performed in our hospital.

Clinical evaluation was carried out by using the seven-item bowel function score (BFS) by Rintala^5^. Refer to the previous scale study, the maximum total score is 20, the reference value of normal children is 19 with 18 being the 10th percentile^6,7^. Therefore, in this study, a value of 18 (over 90% of the control group) was used as the lower limit of normality. In addition, the manometric evaluation was performed simultaneously with the high-resolution anorectal manometry. The parameters measured in this study were the sphincter resting pressure, squeeze pressure, and the presence of rectal inhibitory reflex (RAIR). The reference values for the normal sphincter resting pressure were 30 to 60 mmHg, and for the extrusion pressure were 50 to 120 mmHg^8^. The follow-up outcomes were compared with 71 cases of normal perineal fistula (NPF) in the same period.

A standard statistical software package (Windows version 23.0; SPSS Inc., Armonk [NY], US) was used to analyze the data. Continuous variables are expressed as medians (ranges) and compared using Mann–Whitney U test. Use the chi-square test to compare categorical variables. *P* value less than or equal to 0.05 is considered statistically significant.

### Ethics approval and consent to participate

The study was approved by the ethical committee of the Institutional Review Board of Shanghai Jiao Tong University. Informed written consent was obtained from the parents of each participant. Study procedure was carried out in accordance with the approved guidelines.

### Consent for publication

Written informed consent for publication was obtained from all participants. All parents of the study participants gave written consent for their clinical details along with identifying images to be published in this study.

## Results

The time of these children’s diagnosis ranged from 2 days to 10 months after birth (mean [M] = 8.57 ± 107.70). The age at operation ranged from 2.7 to 12.6 months (median [M] = 5.10 ± 2.88). The weight of the patients ranged from 6.2 to 11 kg (M = 7.46 ± 1.59). More than 50% of the SPF patients had a demonstrable cardiac defect (patent foramen ovale, PFO). Additionally, three patients had spine syndrome and two had some degree of urological anomaly (Table 1). All seven children underwent anal anorectoplasty at our hospital. Four patients underwent laparoscopy assisted anorectoplasty (LAARP), and three cases were repaired using modified posterior sagittal anorectoplasty (PSARP). In all seven cases, the proximal dilatation of the bowel was found to be at a high position during the operation. Moreover, we found that the four children who underwent re-operation had serious scars at the original perineal surgery site and surrounding tissues. It was difficult to locate the external sphincter center point by electrical stimulation, and we finally completed the reconstruction surgery after combining with anatomical positioning (Table 2).

**Table 1:** Demographic data with SPF.

Case	Sex	Age at diagnosis	Age at operation	Weight (kg)	Associated anomalies		
					Cardiovascular	Spine	Genitourinary
1	Male	2d	6.2mo	8	–	–	–
2	Male	2d	5.7mo	7	PFO	Tethered cord	Hypospadias Isolated kidney
3	Male	2d	5.1mo	6	PFO	Tethered cord	–
4	Male	2d	4.8mo	6.2	PFO	–	Hypospadias
5	Female	10mo	12.6mo	11	–	Tethered cord	–
6	Male	2d	2.7mo	6.5	PFO	–	–
7	Male	2d	5.0mo	7.5	–	–	–

**Table 2:** Clinical features of patients with SPF.

Case	Surgical approach	Morbidity	Surgical perineal procedures	followed up(y)	BFS
1	LAARP	–	2	4	8
2	LAARP	–	2	4	10
3	LAARP	–	2	3	8
4	PSARP	Mucosal prolapse	1	3	14
5	LAARP	–	1	2.5	18
6	PSARP	Partial bowel obstruction	1	1	18
7	PSARP	–	2	0.5	14

Finally, anorectoplasty was performed successfully in all seven cases. All seven cases were followed up for 0.5–4.0 years (M = 2.57 ± 1.26) after the definitive surgery. Six patients underwent regular outpatient follow-up, while one patient was followed up by telephone. During the follow-up, all seven patients had a seven-item bowel function score (BFS) and high-resolution anorectomanometry. Three cases also underwent contrast enema using barium.

There was no significant difference in the BFS between the different surgical approaches. Three patients who underwent only one operation had no obvious symptoms such as incontinence and constipation. However, the four patients who underwent more than two repair operations demonstrated a lower BFS. Among them, one patient had constipation, while the remaining two exhibited fecal incontinence. Their BFS values were only 8, 10, 8, and 14, which are far below the normal range (Table 2).

Regarding functional evaluation, the rate of constipation (as defined by the Rome III standard) was 14.3% (n = 1) and 7.0% (n = 5) (*p* = 0.44) in the SPF and NPF groups, respectively; however, this was not statistically significant. The rate of soiling (more than once per week) in the SPF group (n = 3, 42.8%) was higher than that in the NPF group (n = 5, 7.0%) (*p* < 0.05). The median BFS in the SPF group was significantly lower (SPF = 12.4, range: 8–18; NPF = 18, range: 16–20, *p* = 0.82), and there were fewer patients with normal BFS in the SPF group (SPF vs NPF = 42.9% vs 86%, *p* = 0.17) (Table 3).

**Table 3:** Comparison of clinical and manometric results of patients between SPF verus NPF.

	SPF (n = 7)	NPF (n = 71)	*p* value
**Functional assessment**			
Constipation	14.3%	7.0%	0.44
Soiling (≥ 1 time per week)	42.8%	7.0%	< 0.05
Median BFS	12.4 (8–18)	18 (16–28)	0.82
% of patients with normal BFS	42.9%	86%	0.17
**Manometric assessment**			
Resting pressure at sphincter (mmHg)	23 (21–26)	34 (13–65)	0.10
% of patients with normal sphincteric resting pressure	0%	72%	< 0.05
Sphincteric squeeze pressure (mmHg)	50 (29–68)	56.3 (13–115)	0.59
% of patients with + RAIR	0%	50.7%	< 0.05

The median sphincter resting pressure in the SPF group was 23 mmHg (range: 21–26 mmHg), whereas in the NPF group it was 34 mmHg (range: 13–65 mmHg) (*p* = 0.10). Compared with the age-matched control group, patients in the NPF group had a normal sphincter resting pressure (SPF vs NPF = 0% and 72%, *p* < 0.05). There was no significant difference between the two groups with regard to the measurement of sphincter compression pressure (SPF = 50 mmHg, range: 29–68 mmHg; NPF = 56.3 mmHg, range: 13–115 mmHg, *p* = 0.59). However, RAIR was present in 0% (n = 0) and 50.7% (n = 36) of the patients in the SPF and NPF groups, respectively (*p* < 0.05) (Table 3).

## Discussion

Perineal fistula is characterized by a slender perineal tube without nasal tube tissue. The definition of perineal fistula is undisputed and it is one of the most common anorectal malformations in pediatric surgery. However, the special type of perineal fistula (SPF) with a highly dilated colon is very rare and the number of cases reported in the literature is limited to date. In our patients with SPF, the dilated proximal colon was located in front of the iliac crest, which undoubtedly contributed to the complexity of the operation.

In this study, incidence of anorectal malformation with long perineal fistula was 8.97% (7/78). However, there were a few referrals of the long fistula type. So, the incidence should be lower. This incidence may reflect that surgeons often ignore to raise the importance of SPF. About 42.8% of all patients with SPF had spine syndrome of tethered cord which is far higher than NPF.

The diagnosis of perineal fistula is well recognized, and almost all neonates can be diagnosed at birth. A true perineal fistula is a narrow perineal orifice with no visible anal canal, and is located in the midline of the anal sphincter but is not completely surrounded by the sphincter mechanism^9–11^. In boys, the fistula is often found in the normal anal area and base of the scrotum. The fistula in girls is mostly located between the normal anal area and posterior labia. The clinical presentation of SPF is similar to that of a perineal fistula, but the position of the dilated colon is unlikely to be the same as in a normal perineal fistula. However, some surgeons do not perform a preoperative examination to get a correct understanding of the anatomical structure of these anorectal malformations, including the related fistula communication between the rectum and genitourinary tract. In our neonatal unit, all newborns with the anorectal malformation of a perineal fistula were screened by a distal colostogram to confirm the position of the distal rectum. We found that there was a special type of perineal fistula associated with a highly dilated colon. This special type of perineal fistula, if unrecognized preoperatively, may complicate surgical operations to repair anorectal deformities. Therefore, it is imperative that a thorough evaluation (e.g., distal colostogram) be performed to identify an SPF so that it can be correctly treated. There is a lack of consensus in the literature on the best imaging method for accurate anatomical diagnosis at birth. Some authors believe that perineal ultrasound is the best option, while others prefer MRI^12,13^. We believe that no matter which investigation is used to assess the anatomy of the anorectal malformation, preoperative evaluation is essential in children diagnosed with a perineal fistula to identify the possibility of a highly dilated colon. Therefore, we suggest a distal colostogram in the newborn period as well as a pelvic MRI to thoroughly evaluate the anatomy of the anorectal malformation.

Another important decision before surgery is to determine whether to transfer the patient to a center with extensive experience in pediatric surgery. Although there is no consensus on this point, there is strong evidence from adult studies that the surgeon’s technique is independently associated with better outcomes^14–16^. In some local hospitals, surgeons may lack awareness of anorectal malformations. Therefore, we recommend that if there is no pediatric surgeon available at a local hospital, patients should be transferred to a center with extensive pediatric surgery experience.

In most patients born with a SPF, management includes a diverting colostomy. Colostomy is valuable because it can avoid the complications of secondary colon dilation and pseudo-incontinence^17^. In our case series, all patients were managed with a diverting colostomy before the definitive repair.

Although it is the most straightforward anorectal malformation to repair^18,19^, perineal fistula remains a constant point of discussion. With regard to reconstructive surgery, it is essential to select an appropriate surgical approach that ensures optimal defecation function, as blind surgery can seriously affect the long-term defecation function. In this study, we introduced the procedure of correcting this abnormality using a posterior sagittal approach and laparoscopic approach, which can accurately expose the position of the distal rectum. These two approaches can avoid potential complications of a fistula, urethral injury, or intrasphincter anal dislocation.

Our postoperatively routine is to calibrate the anus 3 weeks after surgery and continue to expand it for several months every day. With regard to the postoperative outcome of anorectal malformation treatment, particularly regarding continence, it has been reported that 100% of the patients have bowel control in recto-perineal fistulas and the postoperative pain was also minimal^3^. In our study, 86% of the followed-up NPF patients were continent after the correction surgery at the end of the follow-up period. However, 57.1% of the SPF patients had abnormal bowel function, including constipation and fecal incontinence. Inadequate surgery, coupled with a natural propensity for constipation, can lead to severe fecal impaction that can cause substantial rectal dilation. This dilation can hamper rectal mobility and ultimately affect fecal control. In particular, when surgeons do not perform an adequate preoperative evaluation to accurately understand the anatomy of the anorectal malformation, including an associated fistulous communication between the rectum and the urogenital tract, it may affect the optimal surgical management. Due to this, patients may have to undergo multiple operations and may develop a defecation dysfunction. In our study, children who underwent more than two repair operations had lower BFS than those who had only one operation. In addition, the BFS in SPF patients was lower than that in NPF patients, although the difference was not statistically significant. Therefore, repeated operations and a highly dilated colon may be risk factors for abnormal defecation function in these patients. Although the majority of these patients can be managed with drugs, careful follow-up is vital (as with all patients who have anorectal malformations), so that the patients who need early management of their bowel function can be identified^20^.

## Conclusions

In conclusion, this study reported anorectal malformation with long perineal fistula. Compared with NPF patients, the measurements of BFS and sphincter resting pressure on anorectal manometry were lower in SPF patients. To avoid a misdiagnosis, an anorectal perineal fistula should be examined by a distal colostogram before repair. Selecting an appropriate surgical approach is essential for ensuring optimal defecation function in these children as blind surgery can seriously affect the long-term defecation function.

## Data Availability

The data used to support the findings of this study are included within the article.

## Abbreviations

SPF: Special type of perineal fistula
NPF: Normal perineal fistula
LAARP: Laparoscopy assisted anorectoplasty
PSARP: Posterior aagittal anorectoplasty
BFS: Bowel function score
RAIR: Rectoanal inhibitory reflex
PFO: Patent foramen ovale
