# Author Correction: Anorectal malformation with long perineal fistula: one of a special type

**DOI:** 10.1038/s41598-022-06056-3

**Published:** 2022-01-28

**Authors:** Sen Li, Jun Wang

**Affiliations:** 1 Department of Pediatric Surgery, Xinhua hospital affiliated to Shanghai Jiao Tong University School of Medicine, No. 1665, Kongjiang Road, 200092 Shanghai, China; 2 Department of Pediatric Surgery, Shanghai Children’s Hospital, Shanghai Jiao Tong University, No. 355, Luding Road, 200000 Shanghai, China

Correction to: *Scientific Reports* 10.1038/s41598-021-81056-3, published online 18 January 2021.

In the original version of this Article, Sen Li was incorrectly listed as a corresponding author. The correct corresponding author for this Article is Jun Wang. Correspondence and request for materials should be addressed to wangjun@xinhuamed.com.cn.

In addition, Jun Wang was incorrectly affiliated with ‘Department of Pediatric Surgery, Shanghai Children’s Hospital, Shanghai Jiao Tong University, No. 355, Luding Road, Shanghai, 200000, China’.

Finally, the original version of this Article omitted an affiliation for Sen Li. The correct affiliations are listed below.

Department of Pediatric Surgery, Xinhua hospital affiliated to Shanghai Jiao Tong University School of Medicine, No. 1665, Kongjiang Road, 200092, Shanghai, China

Sen Li & Jun Wang

Department of Pediatric Surgery, Shanghai Children’s Hospital, Shanghai Jiao Tong University, No. 355, Luding Road, 200000, Shanghai, China

Sen Li

The original Article has been corrected.

